# The health status of transgender and gender nonbinary adults in the United States

**DOI:** 10.1371/journal.pone.0228765

**Published:** 2020-02-21

**Authors:** Ethan C. Cicero, Sari L. Reisner, Elizabeth I. Merwin, Janice C. Humphreys, Susan G. Silva

**Affiliations:** 1 Department of Community Health Systems, University of California San Francisco School of Nursing, San Francisco, California, United States of America; 2 Department of Epidemiology, Harvard T.H. Chan School of Public Health, Boston, Massachusetts, United States of America; 3 Department of Pediatrics, Harvard Medical School, Boston, Massachusetts, United States of America; 4 Division of General Pediatrics, Boston Children’s Hospital, Boston, Massachusetts, United States of America; 5 Fenway Health, The Fenway Institute, Boston, Massachusetts, United States of America; 6 School of Nursing, Duke University, Durham, North Carolina, United States of America; 7 College of Nursing and Health Innovation, The University of Texas at Arlington, Arlington, Texas, United States of America; 8 School of Medicine, Duke University, Durham, North Carolina, United States of America; Oregon State University, UNITED STATES

## Abstract

The goal of this exploratory study was to delineate health differences among transgender subpopulations (transgender women/TW, transgender men/TM, gender nonbinary/GNB adults). 2015 Behavioral Risk Factor Surveillance System data were analyzed to compare the health of three groups (TW:*N* = 369; TM:*N* = 239; GNB:*N* = 156). Logistic regression and adjusted odds ratios were used to determine whether health outcomes (fair/poor health, frequent physical and mental unhealthy days, chronic health conditions, and health problems/impairments) are related to group and its interaction with personal characteristics and socioeconomic position. Group was a significant predictor of fair/poor health and frequent mental unhealthy days, revealing significant health differences between the transgender groups. The odds of poor/fair health were approximately 2.5 times higher in TM and GNB adults relative to TW. The odds of frequent mental unhealthy days for TM were approximately 1.5–2 times greater than TW and GNB adults. Among those with health insurance, the odds of fair/poor health for GNB adults was more than 1.5–2 times higher that of TM and TW. Among those without health insurance, TM had over 7 times greater odds of fair/poor health than TW. This study underscores the importance of classifying and examining the health of the transgender population as unique subpopulations, as notable health differences were discovered. TM and GNB adults have significant health concerns, requiring the attention of clinical interventions aimed at promoting health and preventing illness.

## Introduction

The term transgender, or trans, describes an array of individuals whose sex assigned at birth differs from their current gender identity, or one’s sex of being male, female, neither, or both. In contrast, cisgender is an adjective used to describe individuals with a gender identity that aligns with their sex assigned at birth [1]. Transgender individuals who were assigned male at birth and currently identify as women or female are transgender women (TW); previously referred to as “male-to-female” [1]. Those assigned female at birth and currently identify as men or male are transgender men (TM); previously referred to as “female-to-male” [1]. People who self-identify as gender nonbinary (GNB) are members of the transgender population who have a gender identity that is inconsistent with cultural and social expectations (e.g., gender nonconforming, genderqueer, or having a gender outside the traditional female-male binary) are also members of the transgender population [2]. Some, but not all, trans people pursue gender-affirming medical interventions such as cross-sex hormones and/or gender-affirming surgeries and other body modifications [3, 4]. In the United States (U.S.), the transgender population is comprised of an estimated 1.4 million TW, TM, and GNB adults, which is based on data from the Centers for Disease Control and Prevention’s (CDC) 2014 Behavioral Risk Factor Surveillance System (BRFSS), the first time a national population-based survey provided measures to identify transgender participants [5–7]. For this study, we use the term transgender to represent the heterogeneity of individuals illustrating the diversity of gender identities, expressions, and roles found within transgender and gender nonbinary communities.

The transgender population experience health disparities and social inequalities associated with personal characteristics (age, race/ethnicity, sexual orientation, and marital status) and socioeconomic position (educational attainment, employment status, income, and health insurance) [2, 8–10], factors known to impact the health of the general public [11–13]. To date, the majority of transgender health research has taken a narrow view of health by focusing on mental health [14, 15], cross-sex hormone therapy [16, 17], and absence of engagement in health-harming behaviors [18–20]. Physical health has most often been examined in relation to HIV/AIDS in transgender health research [21–23] or gender-affirming medical interventions [17, 24, 25]. Despite limitations, these studies contribute to our limited understanding of transgender health and offer as evidence that transgender subgroups have distinct health risks and outcomes. Findings also indicate that transgender adults have unfavorable risk factors [2, 10, 26], including disproportionate levels of discrimination in healthcare settings [26–28] and worse health than their cisgender peers [29, 30]; however, health-related knowledge representing the diversity of gender identities and expressions found within the transgender population is sparse, leaving many communities underrepresented and unexplored [31–33].

Contemporary, empirically-based knowledge of transgender health is scant and lacks understanding of physical health, health problems or impairments, chronic health conditions, and the impact of individual factors known to affect health outcomes in other vulnerable populations such as socioeconomic position and sexual orientation [34, 35]. Despite a growing body of health-related literature, additional research is needed to advance our understanding of health among transgender subgroups such as TW, TM, and GNB adults. Guided by the socioecological model and the intersectionality framework, this study sought to address these knowledge gaps. Suggested by the National Academy of Medicine [36] and demonstrated by previous transgender health research [27, 37], both intersectionality [38] and the socioecological model [39] serve as a critical lens to understand the health of transgender people and how their health outcomes are influenced by individual factors. Intersectionality provides a means to understand how multiple marginalized social identities intersect creating unique experiences reflecting the systems and structures of oppression and privilege [38]. The goal of the intersectionality framework is to outline that belonging to a group with social disadvantage and relegation to another will produce different lived experiences at the individual and institutional level [38]. Whereas, the socioecological framework describes the influence of multiple environmental factors and policy context that can influence an individual’s health and health behaviors [39]. It was originally constructed to inform the development of comprehensive interventions, targeting the multifactorial mechanisms impacting health behaviors at the individual level [39]. Three implicit assumptions of the framework include 1) an individual’s health and well-being are influenced by multiple factors within their social and physical environments; 2) in addition to external factors, an individual’s health status is influenced by their own personal characteristics; and 3) to promote health and well-being one must consider the dynamic relationships and interactions between an individual and their social and physical environments, versus concentrating only on their personal characteristics [39].

Together, the frameworks posit that the overarching influence of structural determinants such as local, state, and national laws and policies affect the social, community, and institutional environments where a transgender person lives, works, and studies; consequently and distinctly influencing how each transgender subgroup copes with stress, engages in health-related behaviors, and accesses resources directed at improving their health and quality of life. By applying the frameworks to this study, we anticipate there to be health differences among the three transgender groups, and personal characteristics associated with one’s minority status (*e*.*g*., sexual orientation) will be contributing factors in the health of transgender adults.

The overall goal of this exploratory study was to increase understanding of the relationships among individual factors (personal characteristics and socioeconomic position) and health status in three transgender study groups (TW, TM, GNB adults). The specific aim was to determine whether health status outcomes are related to study group (TW, TM, GNB adults) and its interaction with personal characteristics and socioeconomic position.

## Methods

This exploratory study used a descriptive and observational study design to perform a secondary analysis of publicly available data from the CDC’s 2015 BRFSS. This secondary analysis compared three transgender groups (TW, TM, GNB adults), individual factors, and health status outcomes. Individual factors included personal characteristics (age, race/ethnicity, sexual orientation, and marital status) and socioeconomic position (educational attainment, employment status, annual household income, and health insurance status). Health status outcomes included measures of self-rated general health (fair/poor health), health-related quality of life (frequent physical and frequent mental unhealthy days), two or more chronic health conditions, and three or more health problems or impairments. Three additional covariates were included in the analytic models to account for the influence of state-level factors (discriminatory laws/policies and percent voting Republican) and the effects of seasonal variation during BRFSS data collection. Institutional review board exemption was obtained from the Duke University School of Nursing.

### 2015 behavioral risk factor surveillance system database

The BRFSS, an annual cross-sectional telephone survey conducted by the CDC and implemented in all U.S. states and participating U.S. territories, collected data on the health and health behaviors of non-institutionalized adults, aged 18 years or older, who resided in the U.S. [40]. Interviews were conducted at the state-level where data collection was a probability sample of all households with landline and cellular telephones [40]. All BRFSS interviews began with a core set of standardized questions and were followed by optional modules and state-added questions. Data collected from the optional modules or state-added items did not affect the interview schedule [40].

In 2015, an optional module assessing gender identity and sexual orientation was available for use for the second consecutive year [41]. For the gender identity portion of this module, participants were asked, “Do you consider yourself to be transgender?”, and if affirmed they were asked, “Do you consider yourself to be male-to-female, female-to-male, or gender nonconforming?” [42, p69]. Twenty-two states (Colorado, Connecticut, Delaware, Georgia, Hawaii, Idaho, Illinois, Indiana, Iowa, Kansas, Maryland, Massachusetts, Minnesota, Missouri, Nevada, New York, Ohio, Pennsylvania, Texas, Virginia, West Virginia, and Wisconsin) adopted this module and collected data on 764 transgender adults [43].

Of important note, if the BRFSS participants asked about the term *gender nonconforming*, the BRFSS surveyor would explain that gender nonconforming people “do not identify only as a man or only as a woman” [42, p70], a definition that accurately describes gender nonbinary persons [44]; whereas, gender nonconforming “describes a person whose gender expression differs from a given society’s norms for males and females” [45, p5]. Given this distinction, this study will use the term *gender nonbinary* in lieu of *gender nonconforming*.

### Analysis sample

Responses to the BRFSS gender identify measure were used to classify transgender participants as TW, TM, or GNB adults. These three transgender identities represent three distinct levels of study group, the factor of main interest; the analytic sample consisted of 369 TW, 239 TM, and 156 GNB adults.

### Measures

Table 1 details the study variables, their descriptions and coded values; bold indicates the key study variables and health status outcomes evaluated in the analytic models. The five health status outcomes explored were fair/poor health, frequent physical unhealthy days (5 or more days within the last 30 days), frequent mental unhealthy days (5 or more days within the last 30 days), two or more chronic health conditions, and three or more health problems or impairments.

**Table 1:** Coding and description for study variables: Study populations, individual and state-level factors, seasonality, and health status outcomes.

Category	Variable	Coding and Description
*Study populations*	**Study group**	[1 = transgender woman (TW), 2 = transgender man (TM), 3 = gender nonbinary (GNB), 4 = cisgender men (CM), 5 = cisgender women (CW)] *Do you consider yourself to be transgender*? *If yes*, *do you consider yourself to be*: *male-to-female*, *female-to-male*, *or gender nonconforming*? *If no*, *BRFSS surveyor determined sex by vocal timber*.
*Individual factors*		
Personal characteristics	**Age**	Age, in years. Range: 18–79, ≥80 (reference:^a^ descending from oldest to youngest in regression models)
	Race/ethnicity	1 = White, NH; 2 = Black, NH; 3 = Hispanic; 4 = other race/ethnicity, NH (American Indian/Alaska Native; Asian only, NH; Native Hawaiian/other Pacific Islander only, non-NH; other race only, NH; or multiracial, NH)
	**Racial/ethnic minority**	1 = racial/ethnic minority, 0 = racial/ethnic majority (reference) *Racial/ethnic minority = Black*, *NH; Hispanic; other race/ethnicity*. *Racial/ethnic majority = White*, *NH*
	Geographic classification	[0 = suppressed, 1 = not in MSA, 2 = in MSA] *BRFSS used participant zip code to determine if in/out of a MSA*, *or BRFSS suppressed for participant confidentiality*.
	Sexual orientation	[1 = straight/heterosexual, 2 = lesbian or gay, 3 = bisexual, 4 = other, 5 = don’t know/not sure] *Do you consider yourself to be*: *straight*, *lesbian or gay*, *bisexual*, *other*, *or don’t know/not sure*?
	**Sexual minority**	1 = lesbian or gay, bisexual, other, and don’t know/not sure; 0 = heterosexual (reference)
	Marital status	1 = married, 2 = divorced, 3 = widowed, 4 = separated, 5 = never married, 6 = member of an unmarried couple
	**Unmarried**	1 = divorced, widowed, separated, never married, 0 = married and member of an unmarried couple (reference)
Socioeconomic position	Educational attainment	[1 = some high school or less, 2 = high school/GED graduate, 3 = some college, 4 = college graduate] *Highest grade or year of school completed*.
	**High school graduate or less**	1 = high school/GED graduate or less, 0 = some college or college graduate (reference)
	Employment status	1 = employed for wages/self-employed, 2 = out of work, 3 = homemaker, 4 = student, 5 = retired, 6 = unable to work
	**Not working**	1 = out of work, homemaker, student, retired, unable to work; 0 = employed, (reference)
	Annual household income	[1 = <$20K, 2 = $20K to <$50K, 3 = ≥$50K] *Annual household income from all sources*.
	**Low income**	1 = <$20K 0 = ≥$20k (reference)
	Health insurance status	[1 = yes, 0 = no] *Do you have any kind of health care coverage*, *including health insurance*, *prepaid plans such as HMOs*, *government plans such as Medicare*, *or Indian Health Service*?
	**Uninsured**	1 = yes, 0 = no (reference)
*State-level factor*	**Discriminatory laws/policies**^b^	[1 = yes, 0 = no (reference)] *Does state have any laws or policies that harm or deliberately targets transgender people*?
	**Percent voting Republican**^c^	[Range: 27.84%–62.30%] *Proportion of voters who voted for the Republican candidate in the U*.*S*. *2012 presidential election*.
*Seasonality*	**Winter/Fall**	[1 = yes, 0 = no (reference)] *BRFSS data collection occurred during Winter/Fall (September–February)*.
*Health status outcomes*		
Self-rated general health	**Fair/poor health**	[1 = fair or poor self-rated general health, 0 = excellent, very good, or good self-rated health] *Would you say your general health is excellent*, *very good*, *good*, *fair*, *or poor*?
Health-related quality of life	Physical unhealthy days	Range: 0–30 days. *Thinking about your physical health*, *which includes physical illness and injury*, *for how many days during the past 30 days was your physical health not good*?
	**Frequent physical unhealthy days**	1 = ≥5 physical unhealthy days during the past 30 days, 0 = 0–5 days
	Mental unhealthy days	Range: 0–30 days. *Thinking about your mental health*, *which includes stress*, *depression*, *and problems with emotions*, *for how many days during the past 30 days was your mental health not good*?
	**Frequent mental unhealthy days**	1 = ≥5 mental unhealthy days during the past 30 days, 0 = 0–5 days
Chronic health conditions^d^	≥**2 chronic health conditions**	[1 = ≥2 chronic health conditions, 0 = <2 chronic health conditions] *Based on the following 9 individual chronic health conditions*: *heart disease (heart attack/myocardial infarction*, *angina*, *or coronary health disease); kidney disease*, *diabetes; COPD*, *emphysema*, *or chronic bronchitis; asthma; stroke; cancer (skin and any other type of cancer); a form of arthritis*, *rheumatoid arthritis*, *gout*, *lupus*, *or fibromyalgia; or a depressive disorder including depression*, *major depression*, *dysthymia*, *or minor depression*
Health problems or impairments	≥ **3 health problems or impairments**	[1 = ≥3 health problems or impairments, 0 = <3 health problems or impairments] *Based on the following 7 individual health problems or impairments*: *limited in any way in any activities because of physical*, *mental*, *or emotional problems; uses special equipment (cane*, *a wheelchair*, *special bed*, *or special telephone);blind or have serious difficulty seeing*, *even with glasses; serious difficulty walking or climbing; difficulty dressing or bathing; serious difficulty concentrating*, *remembering*, *or making decisions because of a physical*, *mental*, *or emotional condition; or difficulty doing errands alone because of a physical*, *mental*, *or emotional condition*

Description of variables provided in italics. Bold items represent key study measures used in analytic models. BRFSS: Behavioral Risk Factor Surveillance System; COPD: Chronic Obstructive Pulmonary Disease; MSA: Metropolitan Status Area; NH: Non-Hispanic.

^a^Reference = reference group for the logistic regression models.

^b^Discriminatory laws/policies from Movement Advancement Project.

^c^Percentage voting Republican data from the Federal Election Commission.

^d^BRFSS participants are asked if a doctor, nurse, or other health professional had ever told them that they had the chronic health condition.

Individual factors (personal characteristics and socioeconomic position measures) were evaluated because they reflect the social and economic factors that influence health-related beliefs, behaviors, and outcomes [11, 46]. With the inclusion of individual factors, this study allowed us to examine study group differences in health status after taking into account the influence of individual factors, as well as evaluate the influence of individual factors and their interactions with study group on health status.

State-level characteristics and seasonality were included as covariate to adjust for effects of the variables on the health outcomes [47–49]. At the state level, publicly data from the Movement Advancement Project [50] were used to indicate if the state where the BRFSS participant resides had any laws or policies that harmed or deliberately targeted transgender people, and publicly available data from Federal Election Commission [51] were accessed to determine the percentage of voters who voted for the Republican candidate in the U.S. 2012 presidential election. Both variables have been associated with health-related outcomes in both transgender and cisgender populations [48, 49, 52], and were included in the models to account for the influence of from these two state-level predictors of health. Previous studies using BRFSS data showed seasonal variations in health-related quality of life such that Winter months adversely impacted physical health, and Fall months negatively influenced mental health [47]. As such, seasonality, in this study, reflects if BRFSS data collection occurred during Winter/Fall months.

### Data analysis plan

Descriptive statistics were used to detail the study variables and health status outcomes for each of the three study groups. Non-directional statistical tests were performed using SAS 9.4.1^®^ with the level of significance set at 0.05 for each test and *a posteriori* contrasts. The level of significance was not adjusted for multiple outcomes and tests due to the exploratory nature of this study.

For all statistical analyses, only cluster and strata BRFSS data were incorporated to account for the complex survey design; sampling weights were excluded. The decision to exclude sampling weights in our analyses emerged from the discovery of measurement error engendered by BRFSS data collection procedures that produced a discordance between BRFSS surveyor-assigned sex and self-reported gender identity [53, 54]. As a result, 74% of TW were classified as cisgender men and 66% of TM were classified as cisgender women [54]. It is not possible to determine the classification accuracy for GNB adults because they do not identify as a man or woman. The classification accuracy of the transgender BRFSS participants was essential for the sex-specific raking algorithm BRFSS used to create sampling weights [40]. Due to the misclassification of the majority of TW and TM, inaccurate and problematic sampling weights were created [54]. The application of BRFSS sampling weights has the potential to negatively impact the validity of statistical conclusions derived from these data [54].

#### Study groups: Individual factors and health outcomes

Bivariate logistic regression for binary measures and one-way analysis of variance methods for continuous measures were performed to test for study group differences in personal characteristics, socioeconomic position, state-level factors, seasonality, and health outcome. Analysis of variance procedures were conducted using a General Linear Model (GLM) approach due to unequal sample sizes of the three study groups. For binary variables, *a posteriori* pairwise contrasts of the study groups were conducted when a significant overall study group effect was detected. For each contrast, the effect size and its 95% confidence interval (CI) were estimated to address magnitude of effect and clinical significance.

#### Study groups and health outcomes: Multivariable prediction models

Multivariable logistic regression models were utilized to determine the influence of study group, individual factors, and study group and the individual factor interaction on health status outcomes, covarying for state-level factors and seasonality. A separate analysis was conducted for each health status outcome. Each comprehensive model was then reduced to a final model using an iterative backward elimination variable selection method, whereby the least significant term was omitted from model one at a time until a final model was achieved. The final model included: (a) study group; (b) significant individual factors; (c) significant study group-by individual factor interaction term; and (c) significant state-level and/or seasonality covariates. Study group and components of a significant interaction were retained regardless of statistical significance. Statistical significance was set at 0.05 for each predictor term in the comprehensive and reduced model. Adjusted odds ratios (aOR) and their 95% CI were used to estimate effect size for significant predictor terms and the mean probabilities for subgroups of a significant interaction were obtained. *A posteriori* pairwise contrasts of the study groups were conducted when a significant overall study group effect or within each level of the individual factor when a significant interaction was detected.

## Results

### Study groups: Individual factors and health outcomes

Descriptive statistics for study measures and the results of the bivariate analyses are provided for the three study groups in Tables 2 and 3. The study groups differed significantly on being a sexual minority, uninsured, and the percentage of voters who voted for the Republican candidate in the U.S. 2012 presidential election (Table 2 and S1 Table). Compared to TM, TW were nearly 1.5 times more likely to identify as a sexual minority. GNB adults had approximately 1.75 higher odds of identifying as a sexual minority relative to TW and over 2.6 higher odds than TM. Relative to TW, the odds of being uninsured were 1.85 higher in TM and approximately 1.7 times greater for GNB adults. TM were 10% more likely to be uninsured than GNB adults.

**Table 2:** Bivariate model results: Study group, individual and state-level factors, and seasonality.

Variable	TW (*N* = 369)	TM (*N* = 239)	GNB (*N* = 156)	*P*
*Personal characteristics*				
**Age**, in years (Mean ± SEM)^a^	53.89 ± 0.89	54.55 ± 1.15	52.77 ± 1.53	0.6467
Race/ethnicity	369	239	156	**–**
White, non-Hispanic (NH)	263 (71.27)	166 (69.46)	104 (66.67)	
Black, NH	29 (7.86)	20 (8.37)	11 (7.05)	
Hispanic	32 (8.67)	33 (13.81)	18 (11.54)	
Other racial/ethnic minorities, NH	45 (12.20)	20 (8.37)	23 (14.74)	
**Racial/ethnic minority**	106 (28.73)	73 (30.54)	52 (33.33)	0.5051
Sexual orientation	366	234	153	**–**
Heterosexual	296 (80.87)	202 (86.32)	108 (70.59)	
Lesbian or gay	13 (3.55)	9 (3.85)	9 (5.88)	
Bisexual	39 (10.66)	13 (5.56)	29 (18.95)	
Other sexual orientations	12 (3.28)	4 (1.71)	5 (3.27)	
Don’t know/not sure	6 (1.64)	6 (2.56)	2 (1.31)	
**Sexual minority**	70 (19.13)	32 (13.68)	45 (29.41)	**0.0001**
Marital status	365	236	156	–
Married	178 (48.77)	114 (48.31)	64 (41.03)	
Divorced	58 (15.89)	27 (11.44)	19 (12.18)	
Widowed	26 (7.12)	38 (16.10)	23 (14.74)	
Separated	7 (1.92)	8 (3.39)	8 (5.13)	
Never married	87 (23.84)	44 (18.64)	38 (24.36)	
Member of an unmarried couple	9 (2.47)	5 (2.12)	4 (2.56)	
**Unmarried**	178 (48.77)	117 (49.58)	88 (56.41)	0.1869
*Socioeconomic position*				
Educational attainment	368	238	156	**–**
Some high school or less	44 (11.96)	42 (17.65)	15 (9.62)	
High school graduate	142 (38.59)	81 (34.03)	59 (37.82)	
Some college	106 (28.80)	58 (24.37)	41 (26.28)	
College graduate	76 (20.65)	57 (23.95)	41 (26.28)	
**High school graduate or less**	186 (50.54)	123 (51.68)	74 (47.44)	0.6574
Employment status	366	234	154	**–**
Employed	161 (43.99)	105 (44.87)	65 (42.21)	
Out of work	27 (7.38)	10 (4.27)	10 (6.49)	
Homemaker	13 (3.55)	14 (5.98)	8 (5.19)	
Student	20 (5.46)	11 (4.70)	8 (5.19)	
Retired	103 (28.14)	65 (27.78)	46 (29.87)	
Unable to work	42 (11.48)	29 (12.39)	17 (11.04)	
**Not working**	205 (56.01)	129 (55.13)	89 (57.79)	0.8464
Annual household income	318	203	135	–
<$20K	82 (25.79)	43 (21.18)	34 (25.19)	
$20K to <$50K	114 (35.85)	91 (44.83)	50 (37.04)	
≥$50K	122 (38.36)	69 (33.99)	51 (37.78)	
**Low income**	82 (25.79)	43 (21.18)	34 (25.19)	0.4103
Health insurance status	363	237	154	
**Uninsured**	28 (7.59)	32 (13.39)	19 (12.18)	**0.0283**
*State-level*^a^	369	239	156	**–**
**Discriminatory laws/policies**	100 (27.10)	67 (28.03)	46 (29.49)	0.8236
**Percent voting Republican** (Mean ± SEM)	47.06 ± 0.52	48.87 ± 0.58	46.80 ± 0.92	**0.0382**
*Seasonality*^a^				
**Winter/Fall**	182 (49.32)	108 (45.19)	68 (43.59)	0.3255

Number of participants in study group with data available (*N*, *unweighted*) reported for each variable. For categorical variables, number (*n*), percent (%) of *N*, and *P*-values from logistic regression that accounted for cluster and strata data are provided. Least squares mean ± SD and *P*-values from General Linear Models presented for age and percent voting Republican. Variables included in the analytic models shown in bold. Bold *P*-values indicates statistical significance at the 0.05 level. GNB: gender nonbinary adults; NH: non-Hispanic; SEM: standard error of the mean; TM: transgender men; TW: transgender women.

^a^Age, state-level variables, and seasonality had no missing data.

**Table 3:** Bivariate model results: Study group and health outcomes.

Health status outcome	TW (*N* = 369)	TM (*N* = 239)	GNB (*N* = 156)	*P*
*Self-rated general health*				
Self-rated general health	369	238	156	**–**
Excellent	60 (16.26)	31 (13.03)	17 (10.90)	
Very good	98 (26.56)	75 (31.51)	39 (25.00)	
Good	125 (33.88)	82 (34.45)	50 (32.05)	
Fair	59 (15.99)	27 (11.34)	33 (21.15)	
Poor	27 (7.32)	23 (9.66)	17 (10.90)	
**Fair/poor health**	86 (23.31)	50 (21.01)	50 (32.05)	**0.0199**
*Health-related quality of life*				
Physical unhealthy days	362	227	153	
0 days	225 (62.15)	131 (57.71)	82 (53.59)	
1–5 days	54 (14.92)	39 (17.18)	39 (25.49)	
6–15 days	41 (11.33)	24 (10.57)	13 (8.50)	
16–29 days	11 (3.04)	9 (3.96)	2 (1.31)	
30 days	31 (8.56)	24 (10.57)	17 (11.11)	
**Frequent physical unhealthy days**	93 (25.69)	61 (26.87)	39 (25.49)	0.9246
Mental unhealthy days	360	232	150	
0 days	228 (63.33)	143 (61.64)	95 (63.33)	–
1–5 days	63 (17.50)	38 (16.38)	23 (15.33)	
6–15 days	38 (10.56)	26 (11.21)	15 (10.00)	
16–29 days	14 (3.89)	4 (1.72)	5 (3.33)	
30 days	17 (4.72)	21 (9.05)	12 (8.00)	
**Frequent mental unhealthy days**	81 (22.50)	63 (27.16)	37 (24.67)	0.3809
*Chronic health conditions*	354	231	149	
≥ **2 chronic health conditions**	130 (36.72)	78 (33.77)	61 (40.94)	0.2986
*Health problems or impairments*	366	234	152	
≥ **3 health problems or impairments**	55 (15.03)	40 (17.09)	32 (21.05)	0.1845

Number of participants in study group with data available (*N*, *unweighted*) reported for each variable. For categorical variables, number (*n*), percent (%) of *N*, and *P*-values from logistic regression that accounted for cluster and strata data are provided. Variables included in the analytic models shown in bold. Bold *P*-values indicates statistical significance at the 0.05 level. GNB: gender nonbinary adults; TM: transgender men; TW: transgender women.

The groups differed significantly on only one health status outcome, fair/poor heath (Table 3 and S1 Table). GNB adults reported the highest percent of having fair/poor health and were over 1.5 times more likely to report fair/poor health relative to TW and nearly 1.8 more likely than TM.

#### Study groups and health outcomes: Multivariable models

Each comprehensive multivariable regression model evaluating the influence of study group, individual factors, study group-by-individual factor interactions, state-level factors, and seasonality on each health outcome was reduced to a set of final models (Table 4). Table 5 provides an overview of the significant predictors for each health outcome.

**Table 4:** Final multivariable model results: Health status outcomes.

Health status outcome	*N*	Predictor	F	df	*P*	aOR	CI
*Self-rated general health*							
Fair/poor health	732	Study group	3.31	2	**0.0375**	**–**	**–**
		Sexual minority	6.52	1	0.0110	1.69	1.13, 2.53
		High school graduate or less	4.45	1	0.0354	1.43	1.03, 2.01
		Not working	61.29	1	<0.0001	4.22	2.94, 6.05
		Uninsured	0.95	1	0.3295	0.73	0.38, 1.38
		SG*Uninsured	5.32	2	0.0052	**–**	**–**
*Health-related quality of life*							
Frequent physical unhealthy days	727	Study group	0.53	2	0.5888	**–**	**–**
		Sexual minority	7.87	1	0.0052	1.72	1.18, 2.53
		Not working	49.18	1	<0.0001	3.38	2.40, 4.76
Frequent mental unhealthy days	631	Study group	2.94	2	**0.0540**	**–**	**–**
		Age	22.19	1	<0.0001	0.98	0.97, 0.99
		Sexual minority	8.08	1	0.0047	1.76	1.19, 2.61
		Not working	17.82	1	<0.0001	2.29	1.56, 3.36
		Low income	5.74	1	0.0171	1.62	1.09, 2.41
*Chronic health conditions*							
≥ 2 chronic health conditions	718	Study group	0.78	2	0.4602	**–**	**–**
		Age	67.31	1	<0.0001	1.04	1.03, 1.05
		Unmarried	11.48	1	0.0008	1.70	1.25, 2.32
		Not working	27.93	1	<0.0001	2.39	1.73, 3.31
*Health problems or impairments*							
≥ 3 health problems or impairments	616	Study group	1.14	2	0.3199	**–**	**–**
		Sexual minority	17.49	1	<0.0001	2.72	1.70, 4.35
		Unmarried	9.09	1	0.0028	2.06	1.28, 3.29
		Not working	31.80	1	<0.0001	5.18	2.92, 9.19
		Low income	8.45	1	0.0039	2.07	1.27, 3.38
		Uninsured	4.24	1	0.0401	0.38	0.15, 0.96
		Discriminatory laws/policies	3.85	1	0.0506	1.48	1.00, 2.18

**Fig 1:**
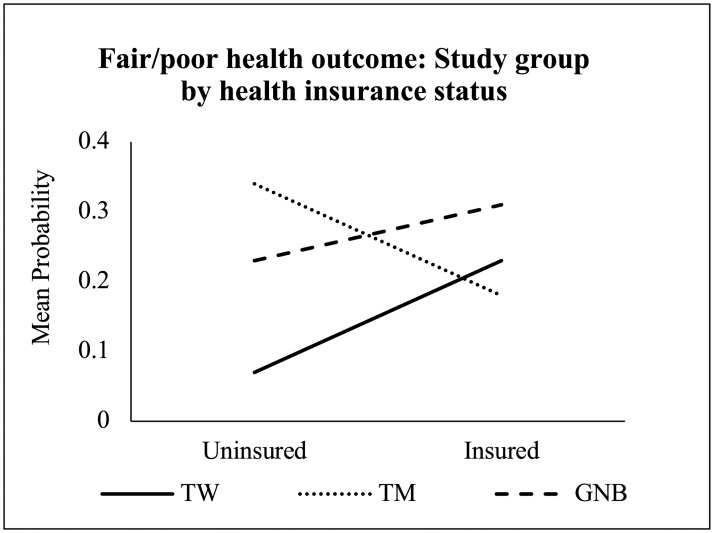
Final multivariable model results: Significant study group interaction for the fair/poor health outcome. GNB: gender nonbinary adults; TM: transgender men; TW: transgender women.

Number of participants (*N*, *unweighted*) included in each model. Bold indicates significant, at the 0.05 level, study group main effects. aOR and their 95% CIs for significant SG main effects and interactions are reported in Tables 4 and 5, as well as Fig 1. Age is in descending order (highest to lowest values). aOR: adjusted odds ratio; CI: confidence interval; df: degrees of freedom; SG: study group.

**Table 5:** Final multivariable model results: Summary of significant predictors for health status outcomes.

Predictor	Health status outcome				
	Fair/poor health	Frequent physical unhealthy days	Frequent mental unhealthy days	≥2 chronic health conditions	≥3 health problems or impairments
Study group	X		X		
Age			X	X	
Racial/ethnic minority					
Sexual minority	X	X	X		X
Unmarried				X	X
High school graduate or less	X				
Not working	X	X	X	X	X
Low income			X		X
Uninsured	X				X
SG* Racial/ethnic minority					
SG*Sexual minority					
SG*Unmarried					
SG* High school graduate or less					
SG* Unemployed					
SG*Low income					
SG*Uninsured	X				
Discriminatory laws/policies					X
Percent voting Republican					
Winter/Fall					

SG: study group.

#### Study group

Study group was a significant predictor of fair/poor health and frequent mental unhealthy days, revealing significant health differences among the transgender groups. The contrasts of the groups (Table 6) indicated that the odds of fair/poor health were approximately 2.3 times higher in TM and over 2.5 times higher in GNB adults relative to TW. TM had nearly 1.5 times higher odds of frequent mental unhealthy days when compared to TW, and 1.7 times more likely to report frequent mental unhealthy days relative to GNB adults.

**Table 6:** Final multivariable model results: *A posteriori* contrasts for study group and health status outcomes.

	Pairwise contrasts		
Health status outcome	TW/TM	TW/GNB	TM/GNB
*Self-rated general health*			
Fair/poor health			
*P* value	**0.0195**	**0.0333**	0.8226
aOR	0.43	0.39	0.92
95% CI	0.21, 0.87	0.17, 0.93	0.42, 1.98
*Health-related quality of life*			
Frequent mental unhealthy days			
*P* value	**0.0431**	0.5613	**0.0324**
aOR	0.67	1.15	1.72
95% CI	0.45, 0.99	0.72, 1.81	1.05, 2.81

Bold indicates statistical significance at the 0.05 level. aOR: adjusted odds ratio; CI: confidence interval; GNB: gender nonbinary adults; TM: transgender men; TW: transgender women.

#### Individual factors

For all health outcomes, employment status was a significant predictor. Not working had higher odds of fair/poor health (aOR = 4.22), frequent physical unhealthy days (aOR = 3.38), frequent mental unhealthy days (aOR = 2.29), two or more chronic health conditions (aOR = 2.39), and three or more health problems or impairments (aOR = 5.18) when compared to employed.

Age was significant predictor of frequent mental unhealthy days and two or more chronic health conditions. For each 10-year increase in age, the odds decreased by 20% for frequent mental unhealthy days and increased by 40% for two or more chronic health conditions.

Sexual orientation was a significant predictor of fair/poor health, frequent physical unhealthy days, frequent mental unhealthy days, and three or more health problems or impairments. Sexual minority had higher odds of fair/poor health (aOR = 1.69), frequent physical unhealthy days (aOR = 1.72), frequent mental unhealthy days (aOR = 1.76), and three or more health problems or impairments (aOR = 2.72) when compared to heterosexual.

Marriage status was a significant predictor of two or more chronic health conditions and three or more health problems or impairments. Being unmarried had greater odds of two or more chronic health conditions (aOR = 1.70) and three or more health problems or impairments (aOR = 2.06) relative to being married.

Educational attainment was a significant predictor of fair/poor health. Having a high school education or less increased the odds of fair/poor health (aOR = 1.43) than those with postsecondary education.

Income was a significant predictor of frequent mental unhealthy days and three or more health problems or impairments. Having a low income increased the odds of frequent mental unhealthy days (aOR = 1.62) and three or more health problems or impairments (aOR = 2.07) than those earning more than $20K.

Health insurance status was a significant predictor of fair/poor health and three or more health problems or impairments. Uninsured had lower odds of fair/poor health (aOR = 0.73) and three or more health problems or impairments (aOR = 0.38) relative to insured.

#### State-level characteristics and seasonality

Discriminatory laws/policies was a significant predictor for three or more health problems or impairments; however, seasonality was not a significant predictor of any health outcome. Living in a state that had a discriminatory law or policy that targets or harms transgender people increased the odds of three or more health problems or impairments (aOR = 1.48) relative to states without such laws or policies.

#### Study group interactions

One study group interaction with an individual factor predicted fair/poor health (Tables 4 and 5). Table 7 summarizes the simple effects results comparing the study groups at each level of the individual factor. Fig 1 provides a graphical representation of this interaction, and S2 Table presents the mean probability for fair/poor health for each interaction subgroup.

**Table 7:** Final multivariable model results: *A posteriori* contrasts for study group interaction for the fair/poor health outcome.

		Uninsured			Insured		
Health status outcome	SG_1_/ SG_2_	aOR	95% CI	*P*	aOR	95% CI	*P*
Fair/poor health	TW/TM	0.14	0.04, 0.53	**0.0038**	1.34	0.87, 2.06	0.1863
	TW/GNB	0.24	0.05, 1.27	0.0938	0.64	0.42, 0.98	**0.0413**
	TM/GNB	1.75	0.41, 7.48	0.4518	0.48	0.29, 0.79	**0.0036**

Bold indicates statistical significance at the 0.05 level. aOR: adjusted odds ratio; CI: confidence interval; GNB: gender nonbinary adults; SG: study group (SG_2_ = reference group); TM: transgender men; TW: transgender women.

The interaction between study group and health insurance was a significant predictor of fair/poor health (Fig 1). The probability of fair/poor health significantly differed among transgender groups when participants reported having health insurance, as well as those without health insurance. GNB adults had the highest mean probability of fair/poor health among participants with health insurance. Among those with health insurance, the odds of fair/poor health for GNB adults was more than 2 times that of TM and over 1.5 times higher than TW. Among those without health insurance, TM had the highest mean probability of fair/poor health and had over 7 times greater odds of fair/poor health than TW.

### Sensitivity analysis

A sensitivity analysis comparing the age and race/ethnicity of the participants within each study group that were included to those excluded, due to missing data, from the final adjusted models revealed no significant differences.

### Power calculation

Power calculations were conducted based on the assumption of a medium effect size represented the smallest clinically meaningful effect (OR of 2.5 or its inverse of 0.40; Cohen *d* equivalent of 0.50). The sample sizes per study group provided at least 80% power for the bivariate analytic models and *a posteriori* contrasts. A sample size of 480 per study group was required to achieve 80% power to test for the relationship between study group on the five health outcomes, after adjusting for the effects of other predictors in the model. Statistical power may not have been adequate for detecting all existing study group differences in the comprehensive multivariable models.

## Discussion

This exploratory and hypothesis generating study examined the health status and individual factors which influence the health of TW, TM, and GNB adults. Further, we sought to highlight the importance of classifying and examining the health of the transgender population as unique subpopulations versus one homogeneous population. Although previous research has identified significant differences in health-related individual factors and health outcomes between transgender and cisgender people [55–58], this study delineated the health differences among transgender subpopulations (groups) in order to identify transgender groups at greatest risk for poor health. Moreover, by acknowledging the heterogeneity of the transgender population, we fill gaps in knowledge about the health of TM and GNB adults, two transgender groups that have been underrepresented in contemporary empirical transgender health research. In addition to this important contribution, our findings suggest that: a) among the transgender groups, there are notable differences in the health of TW, TM, and GNB adults; b) TM have poorer overall health than TW and GNB adults; and c) transgender adults who also identify as a sexual minority have increased odds of having poorer health outcomes than transgender adults who identify as heterosexual. Overall, our findings offer further evidence that both intersectionality and the socioecological model can be used to understand the influence of induvial factors on health status and the differential effects of these correlates across diverse transgender communities.

Our findings provide strong evidence that the health of transgender adults differ among transgender groups. Notably, our findings indicated that GNB adults with health insurance have greater odds of fair/poor health than TW and TM with health insurance. This finding is inconsistent with a large body of evidence linking health insurance with greater healthcare access and improved health outcomes in the general population [59–61], but it is not surprising considering that GNB adults experience higher levels of discrimination in healthcare settings, have more unmet healthcare needs, engage in more health-harming behaviors (i.e., drug/alcohol abuse, smoking, and attempted suicide), and are less likely to have an annual health exam than other transgender groups [27, 28, 62]. Additionally, research exploring the association between discrimination and physical health outcomes in the transgender population are lacking. This represents an important area for future work, especially given that stress is associated with negative, and co-occurring, physical and mental health in other vulnerable populations [63, 64]. Finally, given the disproportionate prevalence of transgender-related discrimination and poorer health experienced by GNB adults relative to TW and TM, health researchers should routinely include transgender-inclusive measures that can identify transgender subgroups and ones that measure transgender-related stigma and discrimination.

Our findings bring attention to the health concerns of TM. We found that among transgender individuals, TM adults may have significant health concerns that require the attention of clinical interventions aimed at promoting health and preventing illness. When factors known to influence health are accounted for, TM had significantly greater odds of fair/poor health when compared to TW and greater odds of frequent mental unhealthy days when compared to both TW and GNB adults. A significant concern about the health of TM in our sample is their lack of health insurance. Not only were TM the study group with the highest percentage of uninsured, TM without health insurance had over a 7-fold increase in the odds of fair/poor health than TW without health insurance. With limited health-related literature about TM, these findings not only contribute to the current knowledge base, but also highlights that health of TM is a research priority.

Study findings suggest that being transgender and a sexual minority adult increase the odds of poorer health outcomes compared to those transgender adults who self-identify as heterosexual, although there were not significant differences across transgender groups. In our sample, sexual minority adults had nearly a 2- to 3-fold increase in poorer health outcomes. This finding underscores the importance of including both sexual orientation and gender identity measures in all health-related research; transgender and gender nonbinary people can be of any sexual orientation and can be attracted to other transgender and/or gender nonbinary people, as well as cisgender individuals. Further, the intersectionality framework indicates the importance of acknowledging the influence of being a member of multiple minority groups on an individual’s lived experiences. Findings from this study elicit the health effects stemming from such lived experiences, particularly for transgender adults who are also identify as a sexual minority person. Often, researchers examining the health of lesbian, gay, bisexual, and transgender (LGBT) individuals group all LGBT people together, compare LGB- to T-identified participants, or do not use measures to identify their transgender participants [65–71].

Our results suggest that discriminatory state-level laws and policies are significant predictors for poorer health outcomes in transgender adults, but not the percentage of state residents voting Republican variable. Excluding the voting party affiliation, these findings are consistent with prior research [52, 72]. One plausible explanation for our findings regarding state-level sociopolitical predictors may stem from the gender identity measure being an optional BRFSS module. States that have incorporated this measure have recognized the importance of collecting health-related data from transgender adults and may therefore have already enacted transgender-protective laws or policies. Without transgender health data collection conducted by all U.S. states and territories, it is difficult to further investigate this inference. Additional insight can be gained if gender identity measures are routinely included in public health surveillance and population-based surveys.

Our findings indicate that significant differences in the health of TW, TM, and GNB adults may exist; although consistent patterns between groups and across outcomes were not apparent. This finding may reflect study limitations associated with BRFSS methodology and the fact that this was a secondary analysis of cross-sectional data. Besides the gender identity module, BRFSS does not include any other survey items regarding transgender health, such as gender-affirming medical interventions (e.g., hormone therapy, surgery). Such interventions have been shown to reduce adverse mental health symptomology and improve the health and well-being of transgender people [3, 73]. However, there are significant barriers to accessing gender-affirming interventions and not all transgender people desire such interventions [2]. Future studies that examine and compare the health status of transgender communities would benefit from additional survey items pertaining to aspects of health-related behaviors known to influence the health of transgender people, such as experiences of discrimination in healthcare settings or uptake of hormone therapy.

Another limitation of this secondary analysis was low statistical power to detect all transgender group differences that may have existed. Results from our final multivariable models suggest that when factors known to influence health are accounted for, GNB adults did not have significantly different health outcomes than other transgender groups, excluding the fair/poor health outcome. In contrast, GNB adults did have the highest percentage, of any study group, of having two or more chronic health conditions, having three or more health problems or impairments, and reporting 30 physically unhealthy days. These conflicting findings may be explained by the small sample sizes of the transgender groups, particularly the GNB group, which was further reduced in size in the multivariable models due to missing data. Future research exploring the health of GNB adults is warranted, and researcher might consider oversampling GNB people in surveys to achieve adequate statistical power.

BRFSS methodology may have contributed to the small GNB sample size, as well as the other transgender groups. For the BRFSS gender identity measure, participants first had to affirm being transgender before they could self-identify as GNB; not all transgender and GNB people use the term transgender when describing themselves [5]. Further, BRFSS is a telephone-based survey, which introduces a social component to gender identity disclosure with the surveyor, including perceived stigma. This may affect any transgender or GNB participant affirming their gender identity. Equally important and specific to GNB adults is BRFSS’s description for gender nonconforming as it does not align with definitions provided by three of the predominant authorities in transgender health; the World Professional Association for Transgender Health, University of California, San Francisco Center of Excellence for Transgender Health, or The Fenway Institute’s National LGBT Health Education Center [3, 44, 45]. The BRFSS gender nonconforming definition reflects a description for a non-binary identity [44]; whereas, gender nonconforming “describes a person whose gender expression differs from a given society’s norms for males and females” [45, p5]. The BRFSS survey measures for gender identity coupled with social stigma associated with transgender identities may undercount the number of self-identified transgender, particularly GNB, respondents.

In spite of the above limitations, our findings make major contributions to understanding the health status of TW, TM, and GNB adults, including components of health that have largely not been studied. Further, our study offers insights to the importance of exploring the health of transgender groups and to the vital importance of including gender identity measures by all researchers, federal, national, and state agencies conducting public health surveillance and population surveys.

## Conclusion

This study, one of the first of its kind, provides a comprehensive depiction of the health status of TW, TM, and GNB adults. Research opportunities remain rich, plentiful, and needed to further identify and prioritize health-related needs and provide the foundation to develop clinical interventions aimed at reducing the burden of illness in the transgender population.

## Supporting information

S1 Table Significant bivariate model results: *A posteriori* contrasts for study group, individual factors, seasonality, and health status outcomes. Bold indicates statistical significance at the 0.05 level. CI: confidence interval; GNB: gender nonbinary adults; OR: odds ratio; TM: transgender men; TW: transgender women.
(PDF)

S2 Table Final multivariable model results: Significant study group interaction for fair/poor health outcome. Least squares means and SEM for estimated probabilities, adjusted for covariates in final multivariate model. GNB: gender nonbinary adults; TM: transgender men; TW: transgender women; SEM: standard error of the mean; SG: study group.
(PDF)
