# Supplementary material for: The health status of transgender and gender nonbinary adults in the United States
Source: PLoS One. 2020 Feb 21;15(2):e0228765. doi: 10.1371/journal.pone.0228765 (PMC7034836; doi:10.1371/journal.pone.0228765)
Supplement: S1 Table — Bold indicates statistical significance at the 0.05 level. CI: confidence interval; GNB: gender nonbinary adults; OR: odds ratio; TM: transgender men; TW: transgender women. (PDF) [file pone.0228765.s001.pdf]

Supporting information, S1

The health status of transgender and gender nonbinary adults in the United States

Ethan C. Cicero[1*], Sari L. Reisner[2-5], Elizabeth I. Merwin[6, 7, #a], Janice C. Humphreys[6], Susan G. Silva[6,8]

S1 Table. Significant Bivariate Model Results: *A Posteriori* Contrasts for Study Group, Individual Factors, Seasonality, and Health Status Outcomes.

	Pairwise contrasts		
Factor	TW/TM	TW/GNB	TM/GNB
Personal characteristic			
Sexual minority			
P value	**0.0444**	**0.0046**	**<0.001**
OR	1.49	0.57	0.38
95% CI	1.01, 2.21	0.38, 0.84	0.24, 0.59
Socioeconomic position			
Uninsured			
P value	**0.0106**	0.0733	0.7214
OR	0.54	0.59	1.11
95% CI	0.33, 0.86	0.34, 1.05	0.63, 1.96
Health status outcome			
Fair/poor health			
P value	0.4891	**0.0245**	**0.0081**
OR	1.14	0.64	0.56
95% CI	0.78, 1.67	0.44, 0.94	0.37, 0.86

Bold indicates statistical significance at the 0.05 level. CI: confidence interval; GNB: gender nonbinary adults; OR: odds ratio; TM: transgender men; TW: transgender women.

[*] Corresponding author. E-mail: ethan.cicero@ucsf.edu; [1] Department of Community Health Systems, University of California San Francisco School of Nursing, San Francisco, California; [2] Department of Epidemiology, Harvard T.H. Chan School of Public Health, Boston, Massachusetts; [3] Department of Pediatrics, Harvard Medical School, Boston, Massachusetts; [4] Division of General Pediatrics, Boston Children's Hospital, Boston, Massachusetts; [5] The Fenway Institute, Fenway Health, Boston, Massachusetts; [6] School of Nursing, Duke University, Durham, North Carolina; [7] College of Nursing and Health Innovation, The University of Texas at Arlington, Arlington, Texas; [#a] College of Nursing and Health Innovation, The University of Texas at Arlington, Arlington, Texas; [8] School of Medicine, Duke University, Durham, North Carolina
