# Supplementary material for: The health status of transgender and gender nonbinary adults in the United States
Source: PLoS One. 2020 Feb 21;15(2):e0228765. doi: 10.1371/journal.pone.0228765 (PMC7034836; doi:10.1371/journal.pone.0228765)
Supplement: S2 Table — Least squares means and SEM for estimated probabilities, adjusted for covariates in final multivariate model. GNB: gender nonbinary adults; TM: transgender men; TW: transgender women; SEM: standard error of the mean; SG: study group. (PDF) [file pone.0228765.s002.pdf]

Supporting Information, S2

The health status of transgender and gender nonbinary adults in the United States

Ethan C. Cicero[1*], Sari L. Reisner[2-5], Elizabeth I. Merwin[6, 7, #a], Janice C. Humphreys[6], Susan G. Silva[6,8]

S2 Table. Final Multivariable Model Results: Significant Study Group Interaction for Fair/Poor Health Outcome.

Health status outcome		Uninsured		Insured	
	SG	Mean	SEM	Mean	SEM
Fair/poor health	TW	0.07	0.04	0.23	0.03
	TM	0.34	0.09	0.18	0.03
	GNB	0.23	0.11	0.31	0.04

Least squares means and SEM for estimated probabilities, adjusted for covariates in final multivariate model. GNB: gender nonbinary adults; TM: transgender men; TW: transgender women; SEM: standard error of the mean; SG: study group.

[*] Corresponding author. E-mail: ethan.cicero@ucsf.edu; [1] Department of Community Health Systems, University of California San Francisco School of Nursing, San Francisco, California; [2] Department of Epidemiology, Harvard T.H. Chan School of Public Health, Boston, Massachusetts; [3] Department of Pediatrics, Harvard Medical School, Boston, Massachusetts; [4] Division of General Pediatrics, Boston Children's Hospital, Boston, Massachusetts; [5] The Fenway Institute, Fenway Health, Boston, Massachusetts; [6] School of Nursing, Duke University, Durham, North Carolina; [7] College of Nursing and Health Innovation, The University of Texas at Arlington, Arlington, Texas; [#a]College of Nursing and Health Innovation, The University of Texas at Arlington, Arlington, Texas; [8] School of Medicine, Duke University, Durham, North Carolina
